# Impact of the COVID‐19 pandemic on people with epilepsy: Findings from the US arm of the COV‐E study

**DOI:** 10.1002/epi4.12637

**Published:** 2022-09-14

**Authors:** Patricia Dugan, Elizabeth Carroll, Jennifer Thorpe, Nathalie Jette, Parul Agarwal, Samantha Ashby, Jane Hanna, Jacqueline French, Orrin Devinsky, Arjune Sen, the COVID‐19 Epilepsy COV‐E Study Group, Jennifer Thorpe, Samantha Ashby, Asma Hallab, Ding Ding, Maria Andraus, Patricia Dugan, Piero Perucca, Daniel Costello, Jacqueline A. French, Terence J. O’Brien, Chantal Depondti, Danielle M. Andrade, Robin Sengupta, Norman Delanty, Nathalie Jette, Charles R. Newton, Martin J. Brodie, Orrin Devinsky, J. Helen Cross, Josemir W. Sander, Jane Hanna, Arjune Sen, Parul Agarwal

**Affiliations:** ^1^ Department of Neurology NYU Grossman School of Medicine New York New York USA; ^2^ SUDEP Action Wantage UK; ^3^ Oxford Epilepsy Research Group, NIHR Biomedical Research Centre, Nuffield Department of Clinical Neurosciences John Radcliffe Hospital Oxford UK; ^4^ Department of Neurology and Department of Population Health Science & Policy Icahn School of Medicine at Mount Sinai New York New York USA

**Keywords:** chronic illness, coronavirus, COVID, epilepsy risk, mental health, seizures

## Abstract

**Objectives:**

As part of the COVID‐19 and Epilepsy (COV‐E) global study, we aimed to understand the impact of COVID‐19 on the medical care and well‐being of people with epilepsy (PWE) in the United States, based on their perspectives and those of their caregivers.

**Methods:**

Separate surveys designed for PWE and their caregivers were circulated from April 2020 to July 2021; modifications in March 2021 included a question about COVID‐19 vaccination status.

**Results:**

We received 788 responses, 71% from PWE (n = 559) and 29% (n = 229) from caregivers of persons with epilepsy. A third (n = 308) of respondents reported a change in their health or in the health of the person they care for. Twenty‐seven percent (n = 210) reported issues related to worsening mental health. Of respondents taking ASMs (n = 769), 10% (n = 78) reported difficulty taking medications on time, mostly due to stress causing forgetfulness. Less than half of respondents received counseling on mental health and stress. Less than half of the PWE reported having discussions with their healthcare providers about sleep, ASMs, and potential side effects, while a larger proportion of caregivers (81%) reported having had discussions with their healthcare providers on the same topics. More PWE and caregivers reported that COVID‐19‐related measures caused adverse impact on their health in the post‐vaccine period than during the pre‐vaccine period, citing mental health issues as the primary reason.

**Significance:**

Our findings indicate that the impact of the COVID‐19 pandemic in the US on PWE is multifaceted. Apart from the increased risk of poor COVID‐19 outcomes, the pandemic has also had negative effects on mental health and self‐management. Healthcare providers must be vigilant for increased emotional distress in PWE during the pandemic and consider the importance of effective counseling to diminish risks related to exacerbated treatment gaps.

**Key Points**

- Healthcare providers must be vigilant for increased emotional distress in people with epilepsy during the pandemic.
- Barriers to healthcare initially related to social restrictions in the COVID‐19 pandemic may be augmented by changing insurance coverage.
- Removing expanded healthcare coverage and telehealth waivers may create additional hindrances to accessing healthcare.

## 1 INTRODUCTION

The ongoing global pandemic caused by the coronavirus disease 2019 (COVID‐19) has presented an extraordinary challenge for health services worldwide, with many healthcare systems forced to make changes at an unprecedented pace. There has been substantial reallocation of resources to care for the large and unpredictable number of the acutely ill. Additionally, efforts to contain viral transmission through, for example, self‐isolation measures, have led to potentially unavoidable deficiencies in the care of chronic illnesses such as epilepsy. In the early months of the pandemic, neurologists and specialist nurses were often redeployed to intensive and acute care units, resulting in less time and fewer staff devoted to the care of people with seizures.^1^ Many hospitals temporarily closed inpatient video‐EEG monitoring units and suspended elective epilepsy surgery, subsequently negatively impacting individuals with drug‐resistant epilepsy.^2^

Responses to COVD‐19 have varied around the world. In the United States (US), 42 states and territories issued mandatory stay‐at‐home orders from March 1 to May 31, 2020.^3^, ^4^ These measures, in addition to drastic social‐distancing guidelines, limited in‐person clinical care. Telemedicine, which can provide a time‐saving and cost‐effective alternative to in‐person clinical encounters for people with epilepsy, was rapidly and widely implemented.^5^, ^6^, ^7^, ^8^ During the first COVID‐19 wave, US insurers moved swiftly to expand coverage to include all telemedicine encounters from home, and states relaxed their licensure requirements to enable care across state boundaries.^9^ The US Department of Health and Human Services also waived enforcement of Health Insurance Portability and Accountability Act regulations to permit commercial audio and video communication and thereby facilitate telemedicine encounters.^9^ Many states subsequently rescinded these waivers as decreed by their respective governors.^10^

The US is currently experiencing its fourth COVID‐19 surge. By January 2022, a year after COVID‐19 vaccines were manufactured, the United States (US) surpassed 65 million COVID‐19 cases, and the death toll had exceeded 850 000 people.^11^

An estimated 1.2% of the US population, approximately 3.4 million people, has epilepsy.^12^ People with epilepsy have experienced worsening of seizures, greater emotional distress, and sleep disruption during this pandemic.^13^, ^14^, ^15^, ^16^, ^17^, ^18^ This reported emotional distress and sleep disruption was similar to the general population, with numbers reported as high as 30% of people citing sleep disturbances, and 71% reporting emotional distress.^19^ A recent meta‐analysis has suggested that people with epilepsy may be at greater risk of poorer COVID‐19 outcomes, particularly disease severity and mortality.^20^ Epilepsy is also a condition that associates with socioeconomic deprivation and health inequity, factors that can be exacerbated during pandemics.^1^

To gain further insights into the challenges people with epilepsy have faced during the pandemic, the COVID‐19 and Epilepsy (COV‐E) Study launched online surveys for people with epilepsy, caregivers, and healthcare workers to assess the impact of the pandemic on health and wellbeing, access to healthcare, and counseling regarding exposure to risk. Responses were submitted from the first wave of the pandemic, through the development and deployment of COVID‐19 vaccines, to the beginning of the fourth surge. The surveys had a global reach across multiple languages; here we present the US data.

## 2 METHODS

### 2.1 Study design

The project, led by UK charity SUDEP Action and University of Oxford (UO), was approved by the UO Ethics Committee, and its design was previously reported.^16^ We used separate online surveys for people with epilepsy (PWE) and primary caregivers of PWE, focusing on quantitative data, with some questions permitting free‐text qualitative responses. The survey was modified and recirculated in March 2021 to include a question about COVID‐19 vaccination status. Data entry was anonymous, and quantitative responses were analyzed in aggregate.

### 2.2 Survey dissemination

Online survey dissemination was an international effort led by SUDEP Action and shared by other epilepsy support organizations including, but not limited to, the BAND foundation, Citizens United for Research in Epilepsy (CURE), Epilepsy Action, Epilepsy Foundation America, Epilepsy Research UK, Epilepsy Society, Epilepsy Sparks, the International Bureau for Epilepsy (IBE), and the International League Against Epilepsy.

### 2.3 Measurements

#### 2.3.1 Demographics

People with epilepsy provided information regarding age, sex, gender, and ethnic background. Caregivers provided the same information about the people with epilepsy for whom they cared.

#### 2.3.2 Epilepsy type/health background

Respondents were queried about epilepsy type, seizure type(s), and frequency. Respondents also provided information about their primary healthcare providers, antiseizure medications (ASM), and access to healthcare in the previous 12 months. We asked about COVID‐19 infections, the need to self‐isolate due to possible exposure and, later, COVID‐19 vaccination status.

#### 2.3.3 Risk factors for epilepsy morbidity and mortality

Respondents answered questions about any changes in sleep quality, seizure patterns, alcohol and drug use, and mental health during the pandemic. They provided information about their living circumstances: whether they lived alone or with someone who could potentially provide first aid.

Respondents also described the counseling they received from their clinicians in the previous 12 months regarding ASM side effects, alcohol or recreational drug use, driving, employment, mental health, pregnancy, safety aids, first aid, the stigma of epilepsy, sudden unexpected death in epilepsy (SUDEP).

#### 2.3.4 Access to healthcare

We investigated the impact of the pandemic on respondents' ability to fill their prescriptions, take ASM on time, communicate with clinicians, and whether there were changes to their scheduled appointments.

#### 2.3.5 Caregiver survey

The caregiver survey emulated the survey for people with epilepsy to determine the impact of COVID‐19 on people with epilepsy from a caregiver's perspective. Caregivers stated the nature of their relationship to the person with epilepsy, for example, parent, spouse, partner, friend, or support worker.

### 2.4 Data analysis

We collated data from the USA from May 2020 to July 2021 and categorized responses into (a) demographics; (b) reported health outcomes; (c) awareness of risk; and (d) access to epilepsy care. Surveys were first analyzed individually, utilizing descriptive statistics and cross‐tabulation of data using tools provided by Jisc, a UK‐based digital infrastructure service. Data were then exported into Microsoft Excel to facilitate cross‐comparison of individual and caregiver surveys.

Additional subgroup analyses examined vulnerability groups, particularly ethnic minorities and those over 60 years. Chi‐squared tests were used to compare responses provided before and after the availability of COVID‐19 vaccines regarding the impact of COVID‐19 on overall health, ability to take medication on time, communicate with their healthcare provider, and scheduling appointments.

## 3 RESULTS

We received 788 responses, 71% from PWE (n = 559) and 29% (n = 229) from caregivers of persons with epilepsy. We received responses from May 2020 to November 2020, and from February 2021 to July 2021.

### 3.1 Population demographics

#### 3.1.1 Sex and gender

Most survey respondents were women (77%, 428/559); 22% were men (125/559), 0.54% identified as non‐binary (3/559), and 0.54% (3/559) did not provide an answer. In the caregiver survey, there was a more even distribution with 49% women (111/229), 50% men (115/229); 0.43% identified as non‐binary (1/229) and 00.87% did not provide an answer (2/229).

#### 3.1.2 Age

Among PWE, most were between 18 and 50 years old (Table 1). PWE under 18 years old were highly represented in the caregiver survey, comprising 53% (122/229) of people cared for by these individuals.

**TABLE 1:** Demographics of the cohort including sex, age, ethnicity, and comorbidities

	People with epilepsy	People with epilepsy (survey completed by caregivers)
n	559	229
Sex		
Female (% within group)	428 (76.5%)	111 (48.5%)
Age group in years (%)		
<18	N/A	122 (53.3%)
18–29	107 (19.1%)	58 (25.3%)
30–39	135 (24.2%)	16 (7.0%)
40–49	118 (21.1%)	12 (5.2%)
50–59	91 (16.3%)	10 (4.4%)
60 and over	101 (18.1%)	8 (3.5%)
Unspecified	7 (1.2%)	3 (1.3%)
Minority ethnic group		
Yes	59 (10.6%)	30 (13.1%)
No	454 (81.2%)	185 (80.8%)
Not sure	23 (4.1%)	8 (3.5%)
Prefer not to say	23 (4.1%)	6 (2.6%)
Comorbidities		
None	177 (31.7%)	0 (0.0%)
Hypertension	83 (14.9%)	13 (5.7%)
Respiratory condition	57 (10.2%)	26 (11.4%)
Memory difficulties	228 (40.8%)	76 (33.2%)
Cardiac condition	35 (6.3%)	8 (3.5%)
Non‐epileptic attacks/dissociative seizures	29 (5.2%)	8 (3.5%)
Mental health	115 (20.6%)	48 (21.0%)
Intellectual disability	30 (5.4%)	94 (41.0%)
Diabetes	18 (3.2%)	1 (0.4%)
Prefer not to say	9 (1.6%)	59 (25.8%)
Other	75 (13.4%)	0 (0.0%)

#### 3.1.3 Ethnicity

Those who self‐identified as minority ethnic groups comprised 11% (89/788) of PWE and those being cared for with epilepsy. Eight responses were omitted from analysis as they identified as belonging to a group that is not regarded as an ethnic minority in the USA as defined by the National Institutes of Health.^21^ Eight percent (60/788) of respondents were unsure or did not answer questions related to ethnicity.

#### 3.1.4 Comorbidities

People with epilepsy and those being cared for with epilepsy reported a high incidence of memory difficulties (39%, 304/788), mental health difficulties (21%, 163/788), and intellectual disability (16%, 124/788). Other conditions included hypertension (12%, 96/788), respiratory issues (11%, 83/788), heart conditions (5%, 43/788), non‐epileptic attacks (5%, 37/788), and diabetes (2%, 19/788). No comorbidities were reported by 22% (177/788).

### 3.2 Exposure to risk during the COVID‐19 pandemic

#### 3.2.1 Health and well‐being

Thirty‐one percent (243/788) of all respondents reported a change in their health or in the health of the person they care for. Overall, 11% (85/788) reported a change in the number, type, or length of seizures. Twenty‐seven percent (210/788) reported increased mental strain, stress, worry, anxiety, or depression. Twenty percent (159/788) reported disrupted sleep patterns. Of all respondents with epilepsy, increased alcohol consumption was reported by 2% (17/788) and increased recreational drug by 1% (9/788).

#### 3.2.2 Access to healthcare

##### Prescriptions

**FIGURE 1:**
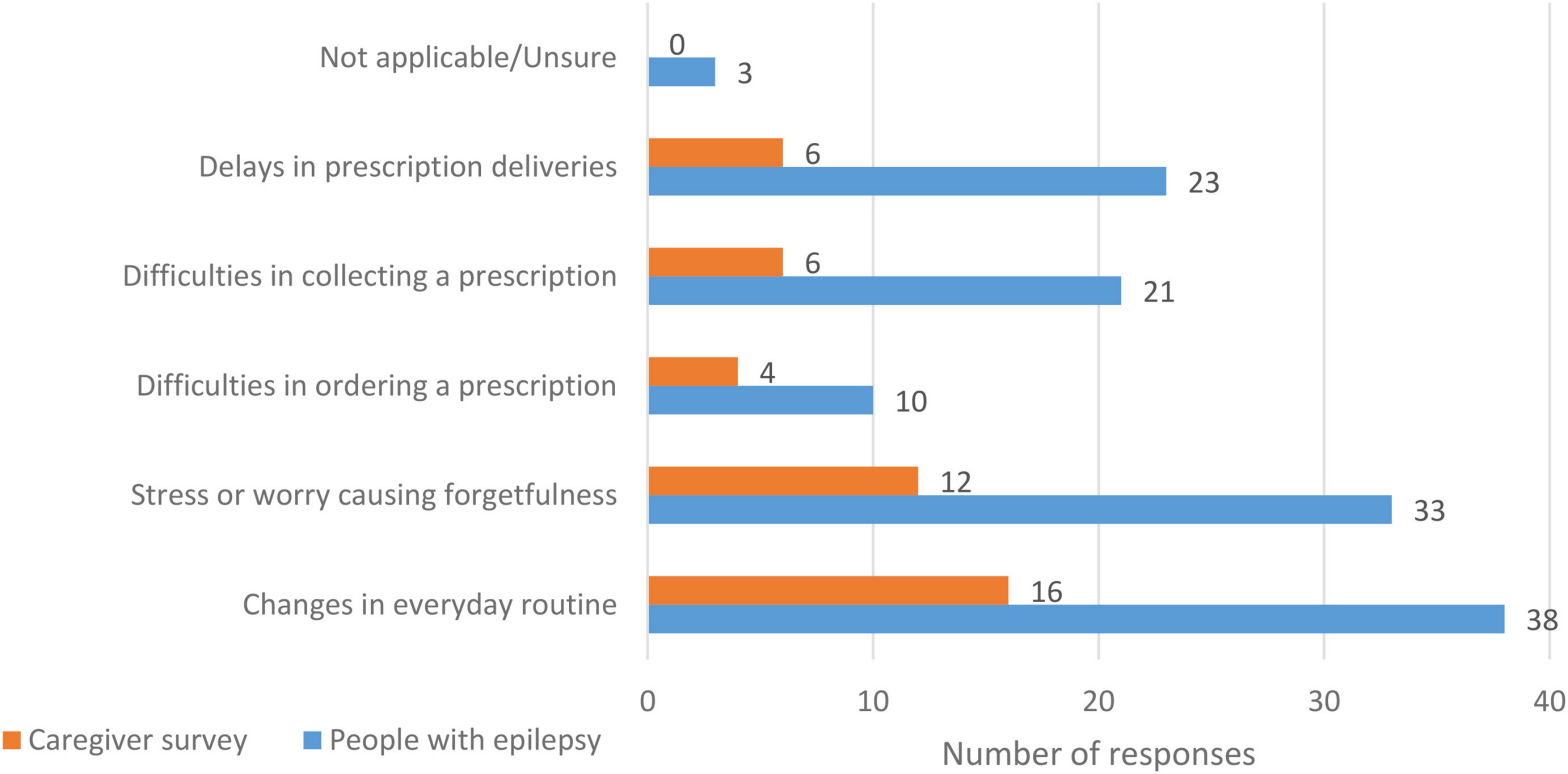
Cited reasons for difficulties adhering to antiseizure medications (ASM) in the caregivers and people with epilepsy (PWE) surveys, respectively. A greater proportion of respondents in the PWE survey reported difficulties in adhering to ASM compared to responses from the caregiver survey. Changes in everyday routine and stress or worry causing forgetfulness were the most commonly cited causes in both groups. Respondents were permitted to provide more than one answer.

Of those taking ASMs (n = 769), 10% reported greater difficulties taking medications on time (78/769), mostly owing to stress and worry causing forgetfulness (7%, 54/769), and changes in daily routine (6%, 45/769). Four percent (29/769) reported delays in prescription deliveries, and 4% (27/769) reported difficulties in collecting prescriptions. Only 2% (14/769; Figure 1) reported problems ordering prescriptions.

##### Medical services

**FIGURE 2:**
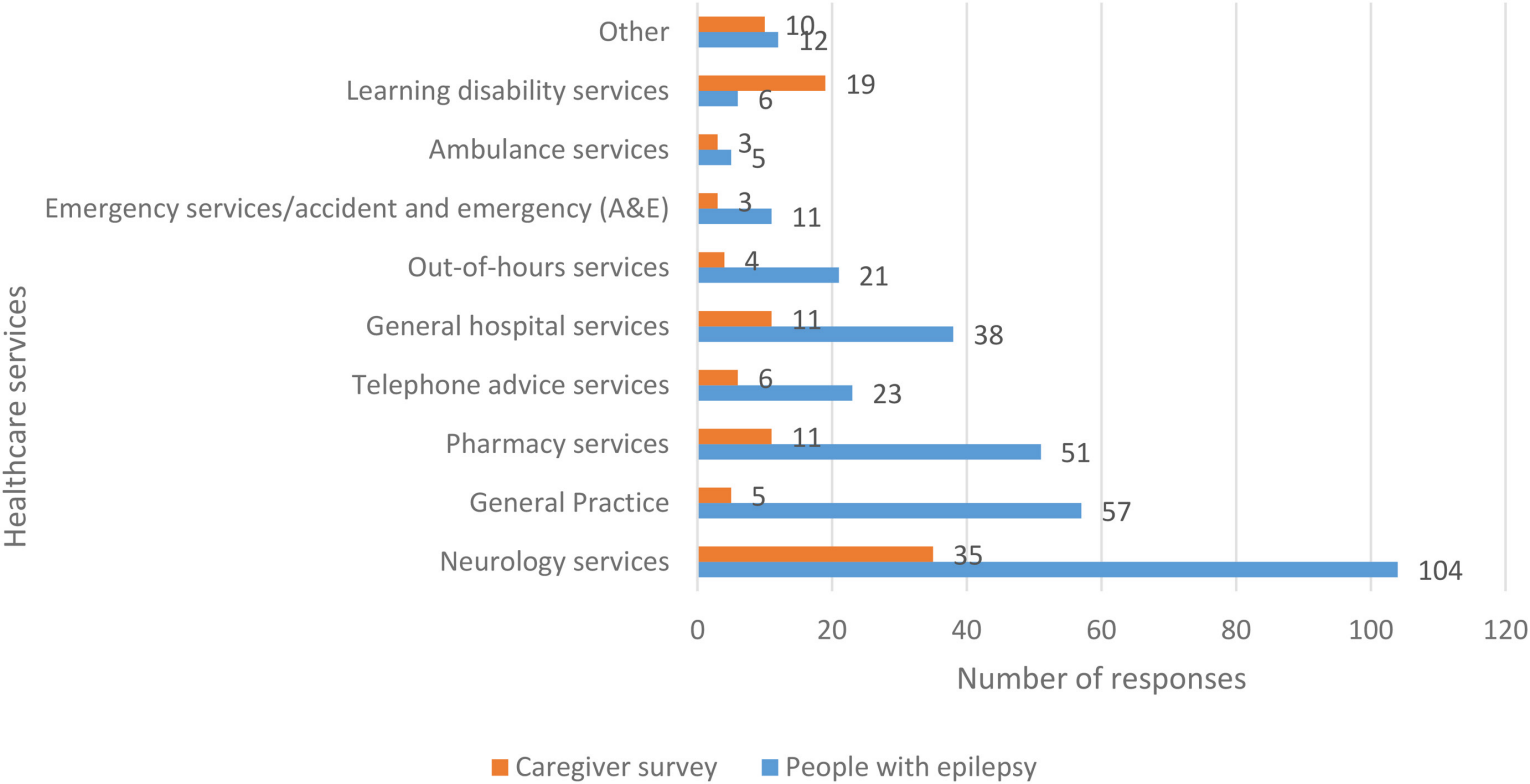
Number of people with epilepsy and caregivers who reported difficulties accessing healthcare services during the COVID‐19 pandemic. Respondents were permitted to provide more than one answer.

Almost a quarter (188/788) of all PWE and caregivers reported difficulty getting health services during the pandemic: 18% (139/788) of respondents reported difficulty reaching Neurology Services; 8% (62/788) had difficulty reaching their General Practitioner and 8% (62/788; Figure 2) had difficulty contacting Pharmacy Services.

##### First aid

Thirty percent (240/788) of PWE were living alone during lockdown. Of the 544 PWE who were co‐habiting, 5.3% (29/544) did not live with anyone who could provide first aid.

##### Emergency care

Twenty‐seven percent (216/788) of all respondents with epilepsy and caregivers reported sustaining injuries or needing emergency care due to epilepsy or related injuries during the 12 months prior to survey completion.

##### Epilepsy services

Fifty‐six percent (443/788) of all epilepsy respondents reported that planned medical appointments were changed. Of these, 60% (266/443) reported that their encounters were rescheduled as telemedicine visits (telephone or video call). Only 4% of all respondents (34/788) reported that their appointments had been canceled and 16% (128/788) were dissatisfied with their scheduling changes.

##### Risk awareness

**FIGURE 3:**
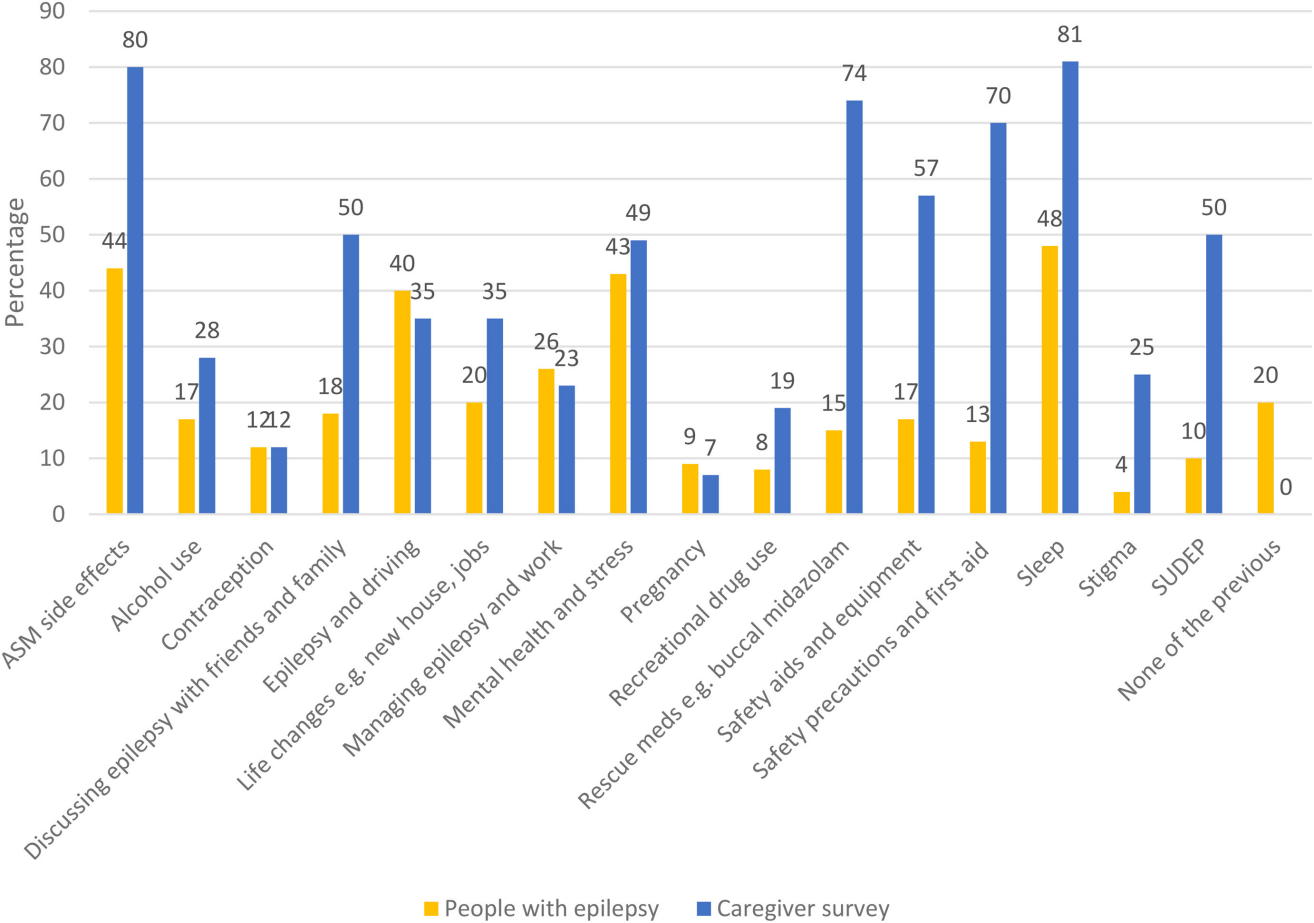
Summary of responses relating to discussion of epilepsy‐related factors. Respondents were asked whether they had discussed specific aspects relating to epilepsy with a clinician in the past 12 months. Less than half of people with epilepsy reported having discussions with their healthcare providers about sleep, antiseizure medications (ASM) and their potential side effects, and other topics related to risk, such as sudden unexpected death in epilepsy (SUDEP) were discussed even less frequently. Many more caregivers reported having had discussions with their healthcare providers on these same topics. This discrepancy might be explained by differences in self‐reported and caregiver‐reported recall.

All respondents with epilepsy and caregivers were asked about counseling they had received from healthcare providers in the previous 12 months. Only 44% percent of people with epilepsy recalled discussions on ASMs and side effects, while 80% of caregivers remembered discussing this with a healthcare provider. Forty‐eight percent of people with epilepsy discussed sleep with healthcare providers, whereas 81% of caregivers reported having had these conversations. Recreational drug use was discussed with 8% of people with epilepsy and 19% of caregivers. Greater differences were reported for SUDEP discussions (10% of people with epilepsy, 50% of caregivers); using safety precautions and first aid (13% of people with epilepsy, 70% of caregivers); rescue medications (15% of people with epilepsy, 74% of caregivers), safety aids and equipment (17% of people with epilepsy, 57% of caregivers). Forty‐three percent of people with epilepsy and 49% of caregivers had discussed mental health and stress with clinicians in the 12 months prior to survey completion (Figure 3).

### 3.3 Special groups

#### 3.3.1 Ethnic minorities

Among the 81 minority ethnic group respondents, most received medical care from neurologists (69%, 56/81) and epileptologists (19%, 15/81). Thirty‐one percent found it difficult to receive health services for their epilepsy during the current pandemic (25/81). Sixty‐four percent (52/81) of these respondents were living with someone during the pandemic although only 57% (46/81) were living with someone who could administer first aid. Thirty‐eight percent (31/81) experienced epilepsy‐related injuries or required emergency care in the prior 12 months.

#### 3.3.2 People with epilepsy aged 60 years and older

One hundred and nine respondents with epilepsy were over the age of 60 years. Forty‐nine percent (53/109) primarily received medical care from neurologists, while 29% and 15% percent primarily saw epileptologists (32/109) and general practitioners (16/109), respectively. Only a quarter (25%, 27/109) of respondents found it difficult to get help during the pandemic. Fifty‐two percent (57/109) of respondents were living with someone during the pandemic and 48% (52/109) were living with someone who could administer first aid. A quarter of respondents (25%, 27/109) had epilepsy‐related injuries or required emergency care in the prior 12 months. The most commonly reported medical comorbidities were hypertension and memory difficulties.

#### 3.3.3 Analysis of responses in the pre‐vaccine and post‐vaccine periods

Forty‐three percent of people with epilepsy (242/599) and 34% (n = 77/229) of caregivers completed this survey from March to July 2021 and provided information about vaccination status. In March 2021, essential workers, adults over 50 years, and those were chronic medical conditions were eligible for the COVID‐19 vaccine; by the end of April 2021, all US adults were eligible.^22^ We found that 64% of people with epilepsy (n = 154/242) and 68% of caregivers or the people under their care (n = 52/77) had received at least 1 dose of a COVID vaccine (Table 2) at that time. Responses to queries regarding access to healthcare or overall health provided between April and November 2020 were compared to responses provided from February to July 2021, after vaccinations were obtainable by the general public (Table 3). Among people with epilepsy, 269 (48%) completed the survey before COVID‐19 vaccines were available and 290 (52%) completed the survey in the post‐vaccine period. Among caregivers, surveys were completed by 149 (65%) during the pre‐vaccine period and 80 (35%) after vaccines were deployed. When asked whether COVID‐19‐related measures had caused any changes to their health, people with epilepsy and caregiver groups reported a greater proportion of affirmative responses (i.e., yes/unsure) in the post‐vaccine period group compared to their respective pre‐vaccine period cohorts: 48% vs 40% among people with epilepsy (*P* = .05), and 54% vs 30% among caregivers (*P* = .0003). A higher proportion of caregivers in the post‐vaccine period also reported difficulties in access to healthcare services as compared to pre‐vaccine period (36% vs 23%, *P* = .03). However, no statistically significant differences were observed between the pre‐ and post‐vaccine period in the responses to questions regarding ability to take medication on time, change in scheduled appointments, or access to healthcare services.

**TABLE 2:** COVID vaccination status of respondents who completed survey during the post‐vaccination era

	People with epilepsy, n = 242	People with epilepsy (survey completed by caregivers), n = 77	Total, n = 319
Received 1 dose	42 (17.4%)	7 (9.1%)	49 (15.4%)
Received 2 doses	112 (46.3%)	45 (58.4%)	157 (49.2%)
Not yet offered vaccine	33 (14%)	5 (7%)	38 (12.0%)
Awaiting first dose	10 (4%)	5 (7%)	15 (4.7%)
Offered vaccine but refused	26 (11%)	13 (17%)	39 (12.2%)
Other	19 (8%)	2 (3%)	21 (6.6%)

**TABLE 3:** Comparison of responses regarding access to healthcare provided pre‐ and post‐vaccine availability

	People with epilepsy			People with epilepsy (survey completed by caregivers)		
	Pre‐vaccine period	Post‐vaccine period		Pre‐vaccine period	Post‐vaccine period	
n	269	290		149	80	
Have recent COVID‐19 measures caused any changes to your/their health?						
Yes/Unsure	107 (40%)	139 (48%)	***P* = .05**	44 (30%)	43 (54%)	***P* = .0003**
No	162 (60%)	151 (52%)		105 (70%)	37 (46%)	
Has COVID‐19‐related disruption made it difficult for you/the person you care for to take medication on time?						
Yes/Unsure	25 (10%)	35 (12%)	*P* = .32	13 (9%)	9 (12%)	*P* = .57
No	231 (90%)	245 (88%)		127 (91%)	68 (88%)	
Have you/the person you care for found it difficult getting help for your epilepsy from health services during the current pandemic?						
Yes/Unsure	61 (23%)	76 (26%)	*P* = .33	34 (23%)	29 (36%)	***P* = .03**
No	208 (77%)	214 (74%)		115 (77%)	51 (64%)	
Have they had planned medical appointments that have been changed?						
No, there has not been any communication about pre‐scheduled appointments	53 (24%)	53 (22%)	*P* = .74	16 (14%)	4 (7%)	*P* = .18
The scheduled appointment was canceled/changed/other reason	170 (76%)	183 (78%)		97 (86%)	52 (93%)	

*Note*: *P*‐values were estimated from chi‐square tests of association. The bold values indicate that they are statistically significant.

Increased mental strain, stress, worry, anxiety, or depression was the primary reason for adverse impact on health in both groups during the pre‐ and post‐vaccine periods.

## 4 DISCUSSION

As the US currently leads the world in the number of COVID‐19 cases and mortalities, the impact of this pandemic on the health and well‐being of people with epilepsy living in America and their caregivers is multifaceted and profound. Our results further demonstrate that the indirect burden of the pandemic is substantial.

While approximately one‐tenth of respondents reported changes in the number, type, or length of seizures, a third of all respondents reported a change in their health or in the health of the person they care for during the pandemic with over a quarter attributing this to increased mental strain, stress, worry, anxiety, or depression. These findings are further supported by a recent study that reported that 57.1% of PWE and 21.5% of caregivers endorsed significant psychological distress.^23^ Deterioration of mood and increased emotional distress in people with epilepsy during the pandemic has also been observed in the UK and Brazilian arms of this study,^16^, ^17^ as well as multiple others.^1^, ^14^, ^15^, ^18^

The US Food and Drug Administration issued the first emergency use authorization for the use of the Pfizer‐BioNTech COVID‐19 vaccine in December 2020 which was soon followed by approval for the Moderna and Janssen (Johnson and Johnson) vaccines.^22^ In March 2021, adults over the age of 50, those with chronic medical conditions, and all essential workers were eligible for the COVID‐19 vaccine; this extended to all adults by the end of April 2021. The availability of these vaccines marked an important milestone and initial optimism for an imminent end to the pandemic.^24^ Analysis of responses provided during the pre‐vaccine and post‐vaccine periods, however, show that a larger proportion of respondents experienced an adverse impact on health as a result of COVID‐19‐related measures in the post‐vaccine period. This applied both to people with epilepsy and caregivers and was, as in the pre‐vaccine cohort, primarily attributed to a decline in mental health and wellbeing. This contrasts with a longitudinal Spanish study evaluating the impact of the pandemic on levels of anxiety, depression, somnolence, and quality of life that showed lower anxiety levels in people with epilepsy.^25^ This was attributed to possibly having more stable living situations during the pandemic. In that study, predetermined severity of epilepsy was not found to be a key factor in COVID‐19‐associated increases in anxiety.^25^

Our post‐vaccine findings may reflect increasing distress related to the surging Delta variant of COVD‐19 as seen in an Australian study that compared the impact of COVID‐19 on behaviors and psychological symptoms of dementia (BPSD) and related caregiver distress during the pre‐COVID‐19 period (January 2018–December 2019) and COVID‐19 period (January 2020–July 2021).^26^ Additionally, despite the safety and tolerability of the COVID‐19 vaccines in people with epilepsy,^27^, ^28^ vaccine hesitancy remains a widespread issue^29^, ^30^ and may have contributed to emotional distress, particularly amid the growing calls for vaccine mandates by mid‐2021.^31^

The proportion of respondents who experienced changes in their scheduled appointments was stable in both groups both during the pre‐vaccine and post‐vaccine periods. Sixty percent of respondents whose appointments were changed indicated that these were converted to telemedicine encounters, reflecting its rapid implementation and the swift adaptation of the medical community in delivering clinical care. Although stay‐at‐home orders had long been lifted and many outpatient practices were once again busy with both in‐person and telemedicine visits, it is possible that many appointments were rescheduled as more and more states rescinded or modified their telehealth waivers.

Analysis of vulnerable groups showed that a similar proportion of respondents aged over 60 years had difficulty accessing health services, had epilepsy‐related injuries, or required emergency care when compared to the entire cohort. A higher proportion of respondents from ethnic minorities, however, found it difficult to receive health services. Ethnic minorities also reported more epilepsy‐related injuries and need of emergency care compared to the entire cohort. In the US where health insurance coverage is not universal, disparities in epilepsy care can associate with race, ethnicity, and socioeconomic status.^32^ These disparities were intensified in the setting of the numerous healthcare disruptions brought about by the pandemic. Our data thus mirror the racial and ethnic disparities in healthcare that have been previously described during the COVID‐19 pandemic with respect to infection, in‐hospital outcomes once admitted, and access to telemedicine.^33^

People with epilepsy can have comorbidities that may place them at greater risk of contracting COVID‐19. The high burden of comorbidities among respondents was reflected in the data presented here. Despite this, less than half of all respondents received counseling on mental health and stress. Less than half of people with epilepsy reported having discussions with their healthcare providers about sleep, ASMs and their potential side effects, and other topics related to risk such as safety precautions and first aid. Rescue medications, recreational drug use, and SUDEP were discussed even less frequently. Many more caregivers reported having had discussions with their healthcare providers on these same topics. This discrepancy might be explained by differences in self‐reported and caregiver‐reported recall; 41% of people with epilepsy reported memory issues as a comorbidity. 41% of those people with epilepsy on whom caregivers reported had intellectual disability and 33% had memory difficulties, requiring caregivers to advocate on their behalf. As the pandemic continues, it remains essential to provide counseling on risk mitigation, particularly to people with epilepsy who have an even greater need for self‐management during this time.^18^, ^34^

Comparing other survey findings from the UK^16^ and Brazilian^17^ arms of the COV‐E study shows, for example, that a third of US, and a quarter of UK, respondents lived alone during the pandemic, while only 9% of Brazilian respondents were solitary, likely reflecting different social and cultural constructs.^17^ Across the three countries, a similar proportion of respondents reported difficulty taking their medications on time (10% US; 13% UK; 11% Brazil). The primary reasons for this lack of adherence differed. Stress and worry causing forgetfulness was cited as the main contributor in the US; changes to routine, particularly the ability to access healthcare services and prescriptions for medications, was the most important factor in the UK; and difficulty obtaining prescriptions and altered or canceled appointments was the principal reason in Brazil. Such discrepancies underscore the need for better country‐contextualized understanding of how COVID‐19 is affecting people with epilepsy and the tailored mechanisms required to mitigate adverse impacts as the pandemic continues to evolve.^34^

## 5 LIMITATIONS

The principal limitations of the COV‐E study have been previously described.^16^ Survey responses in this study were not categorized by state or region although the COVID‐19 pandemic affected different regions of the United States in varying degrees of severity at different time points. As such, US responses may have been influenced by the contemporaneous accessibility of healthcare personnel and resources. Caregivers were instructed to answer on behalf of the person with epilepsy they cared for. All of their survey questions were phrased in a manner to facilitate this (e.g. “Has the person you care for found it difficult getting help for their epilepsy from health services during the current pandemic?”) with the exception of the question, “Have you been offered a COVID‐19 vaccination?” As such, we cannot be certain that their responses reflected their status, or that of the person they care for. Over half of the caregivers surveyed reported caring for a child under the age of 18; potential differences between counseling styles between adult and pediatric neurologists or epileptologists were not investigated. Other study limitations relate to the use of surveys and therefore the possibility of self‐selection bias and potential recall bias. Additionally, the survey was structured such that respondents were not prompted to qualify whether a reported “change” in their seizure burden explicitly referred to worsening or improvement, nor were respondents prompted to describe specific barriers to accessing medical services; further examination of these topics present interesting and important subjects for further investigation.

## 6 CONCLUSIONS

The COVID‐19 pandemic will likely have an enduring impact on how epilepsy care is delivered. PWE should be considered a population at risk during the COVID‐19 pandemic,^20^ due to increased risk of poor COVID‐19 outcomes and the pandemic's effects on mental health and self‐management. In addition to seizures, healthcare providers must be vigilant for increased emotional distress in people with epilepsy during the pandemic and must emphasize the importance of effective counseling to diminish the risks owing to the exacerbated treatment gaps, particularly among vulnerable groups. A multifaceted, person‐centered approach with appropriate use of currently available technologies may achieve these objectives.^16^ In the evolving US COVID‐19 landscape, the barriers to healthcare and communication that initially resulted from social restrictions and resources reallocations may be replaced or augmented by changing insurance coverage. Our findings suggest that removing the mitigations that were initially placed, such as expanded healthcare coverage and telehealth waivers, may have adverse consequences and create additional hindrances to accessing healthcare.

## CONFLICT OF INTEREST

We confirm that we have read the Journal's position on issues involved in ethical publication and affirm that this report is consistent with those guidelines.

## APPENDIX 1 Full list of authors

Jennifer Thorpe^1,2^

Samantha Ashby^2^

Asma Hallab^3^

Ding Ding^4^

Maria Andraus^5^

Patricia Dugan^6^

Piero Perucca^7^

Daniel Costello^8^

Jacqueline A. French^6^

Terence J. O'Brien^7^

Chantal Depondti^9^

Danielle M. Andrade^10^

Robin Sengupta^11^

Norman Delanty^12^

Nathalie Jette^13^

Charles R. Newton^1,14^

Martin J. Brodie^15^

Orrin Devinsky^6^

J. Helen Cross^16,17^

Josemir W. Sander^18,19^

Jane Hanna^2^

Arjune Sen^1^

Parul Agarwal^13^

1. Oxford Epilepsy Research Group, NIHR Biomedical Research Centre, Nuffield Department of Clinical Neurosciences, John Radcliffe Hospital, Oxford OX3 9DU, UK

2. SUDEP Action, 18 Newbury Street, Wantage, Oxfordshire OX12 8DA. UK

3. Department of Nuclear Medicine, Charité – Universitätsmedizin Berlin, Corporate Member of Freie Universität Berlin, Humboldt‐Universität zu Berlin and Berlin Institute of Health, Berlin, Germany

4. Institute of Neurology, Fudan University Huashan Hospital, Shanghai, China

5. Department of Internal Medicine, Neurology Service, Epilepsy Program, Clementino Fraga Filho University Hospital, Federal University of Rio de Janeiro, Rio de Janeiro, Brazil

6. Department of Neurology, NYU Grossman School of Medicine, USA

7. Department of Neuroscience, Central Clinical School, The Alfred Hospital, Monash University, Melbourne, Australia & Departments of Medicine and Neurology, The Royal Melbourne Hospital, The University of Melbourne, Melbourne, Australia

8. Epilepsy Service, Cork University Hospital & College of Medicine and Health, University College Cork, Ireland

9. Department of Neurology, Hôpital Erasme – Université Libre de Bruxelles, Brussels, Belgium

10. Adult Epilepsy Genetics Program, Toronto Western Hospital, University of Toronto, Toronto, Canada

11. The Institute of Neurosciences, Kolkata, India

12. Beaumont Hospital, and School of Pharmacy and Biomolecular Sciences, FutureNeuro Research Centre, Royal College of Surgeons in Ireland, Dublin, Ireland

13. Department of Neurology, Icahn School of Medicine at Mount Sinai, New York, USA

14. University Department of Psychiatry, University of Oxford, UK o Epilepsy Unit, West Glasgow Ambulatory Care Hospital‐Yorkhill, Glasgow, UK

15. UCL NIHR BRC Great Ormond Street Institute of Child Health, London, UK

16. Young Epilepsy, St Pier's Lane, Dormansland, Lingfield RH7 6P, UK

17. UCL Queen Square Institute of Neurology, Queen Square, London WC1N 3BG

18. Chalfont Centre for Epilepsy, Chalfont St Peter SL9 0RJ, UK

19. Stichting Epilepsie Instellingen Nederland (SEIN), Heemstede, the Netherlands

PP is supported by the National Health and Medical Research Council (APP1163708), the Epilepsy Foundation, the Royal Australasian College of Physicians, Melbourne Health, and Monash University. DMA is supported by EpLink, Dravet Syndrome Foundation, McLaughlin grants. NJ is the Bludhorn Professor of International Medicine and her institution receives grant funding from NINDS (NIH U24NS107201, NIH IU54NS100064) and the American Epilepsy Society/NORSE Institute for work she is involved in unrelated to this project. JHC is supported by the National Institute of Health Research (NIHR) Biomedical Research Centre at Great Ormond Street Hospital, NIHR, Engineering and Physical Sciences Research Council, GOSH Charity, Epilepsy Research UK, and the Waterloo Foundation. JWS is based at UCLH/UCL Comprehensive Biomedical Research Centre, which receives a proportion of funding from the UK Department of Health's NIHR Biomedical Research Centres funding scheme. He receives support from the Dr Marvin Weil Epilepsy Research Fund, the Christelijke Vereniging voor de verpleging van Lijders aan Epilepsie, The Netherlands, and the UK Epilepsy Society. AS is supported by the Oxford NIHR Biomedical Research Centre at the John Radcliffe Hospital, UK. All other authors report no conflicts of interest.
